# Pacific meets west in addressing palliative care for Pacific populations in Aotearoa/New Zealand: a qualitative study

**DOI:** 10.1186/s12904-020-00604-2

**Published:** 2020-07-08

**Authors:** Sunia Foliaki, Veisinia Pulu, Hayley Denison, Mark Weatherall, Jeroen Douwes

**Affiliations:** 1 Centre for Public Health Research, Massey University, Wellington Campus, PO Box 756, Wellington, 6140 New Zealand; 2 Department of Medicine, University of Otago, 23A Mein Street, Newtown, Wellington, 6021 New Zealand

**Keywords:** Hospice and palliative care, Pacific communities, Home care, Family obligations to elderly; education

## Abstract

**Background:**

While many Aotearoa/New Zealanders are receiving excellent palliative care the Pacific populations have limited access to available hospice and palliative care services. Little research has been conducted to identify barriers unique to Pacific populations accessing these services. The purpose of this study was to explore key stakeholders’ perspectives on the determinants of low access among Pacific populations to these services.

**Methods:**

Forty-five semi-structured interviews were conducted face-to-face with hospice patients and their families, hospice/health providers and key informants from the Auckland and Wellington region of Aotearoa/New Zealand. The interviews were recorded and transcribed verbatim and a thematic analysis was carried out by identifying, coding and categorising patterns in the data. Identified themes were then discussed further to determine the relevance of the data grouped by theme.

**Results:**

Five interrelated themes affecting access emerged: perception of hospice (often negative) through lack of accurate information, but changing; families’ role to look after their own and sick elderly; hospice experiences; continuity of care in the community and the need for information and communication.

**Conclusion:**

Hospice and associated palliative care services are under-utilised and commonly misunderstood among Pacific populations in Aotearoa/New Zealand. There is active support following appropriate information received, hence the need for community education and culturally appropriate hospice and palliative services. Inadequate inter-professional communication contributes to polypharmacy and inefficiency in continuity of care across all levels. The Pacific individual is one component of a collective that is critical in major decisions in end-of-life and life changing situations. The findings may guide policies and further research to improve Hospice and Palliative services in Aotearoa/New Zealand.

## Background

Palliative care aims to improve the quality of life of patients with life-threatening illness and their families. Although its approach can vary depending on the illness, the main objective is to prevent and relieve suffering through early identification, impeccable assessment, and appropriate management of pain and other dimensions of care, including physical, psychosocial, and spiritual needs [1]. Hospice as an institution provides palliative care services and a philosophy of care that places equal importance to the above dimensions. According to Hospice New Zealand [2], hospices provide palliative care specifically for people whose illness is no longer curable and while the above dimensions of care are a priority, equal importance is placed on cultural needs. Support is also provided for the family before and after the death of their loved ones. Worldwide only one in ten people who need palliative care actually receive it; with the most common illnesses leading to adults requiring palliative care being: cardiovascular diseases (CVD), cancer, and chronic respiratory diseases (CRD) [3]. These are also the leading causes of mortality and morbidity among the Pacific population in Aotearoa/New Zealand (NZ) [4], which grew from just over 2000 in the 1940s, to more than a quarter of a million in 2018, and now make up 8 % of the total population. The Pacific population in NZ generally originate from Samoa, Cook Islands, Tonga, Niuean, Fiji, Tokelau Tuvalu and Kiribati. Common Pacific cultural values that drive and influence Pacific people’s behaviours, decisions, experiences and motivation for change and outcomes include: the central role of the family, kinship, collectivism, relationships, and respect [4].

Amongst the predominantly New Zealand-European population in NZ, most deaths occur at age > 65 years [5], with about 5% of the 65+, and 23% of the 85+ age group living in residential care [6]. A study comparing location of death of those aged over 65 years in twenty one countries reported NZ has the greatest percentage of reported deaths in residential aged care at 38% [7]. The majority of those living in residential care die whilst in care [5], with the physical dependence of these people having significantly increased over the past 20 years with direct implications for greater palliative care needs. In contrast, 44% of all deaths among Pacific populations occur among those under the age of 65 years who are also more likely to die at home or secondary or tertiary care facilities [5]. This raises the importance of not only having culturally appropriate palliative and end-of-life care services but also ensuring services take account of the younger age at death of Pacific populations. In addition to Pacific populations having disability and advanced disease needing palliative care at a younger age, Pacific populations often present late meaning a prolonged economic burden as well as compromising economic productivity due to inability to work and retirement from productive work at a younger age.

Palliative care services, be these provided by either hospice or hospital settings have strong ties to Advanced Care Planning (ACP), with these respective providers visiting patients in their homes [2]. ACP in NZ involves an individual working with his/her healthcare team and important people in his/her life to plan for one’s end-of-life care if he/she can no longer tell them what they want [8]. A guideline prepared for the NZ health care workforce acknowledged the need for community awareness on the roles that palliative care and ACP have in promoting quality care at the end of life through symptom management, social support, community participation, health and death education and reducing harm [9]. In an evaluation of the Advanced Care Planning Programme in NZ, Pacific consumers valued developing their ACPs through in-depth conversations (and preferably in their own homes) with someone they could form a relationship with over time or with someone they had a relationship with [10]. In addition the report found that community meetings are effective at raising consumer awareness and demand for ACP and while some District Health Boards (DHBs) have undertaken a large number of meetings in aged residential care facilities and hospices, and other targeted community groups, DHBs have engaged less with Māori and Pacific communities [10].

Palliative care has not always been responsive to indigenous needs. Among the limited studies on the palliative care needs among specific ethnic minorities were studies in Australia, Canada and Hawaii which highlighted the inadequacies of proximity, resource, historical distrust and the need for culturally safe palliative care in indigenous populations [11–14]. There have been limited studies on the palliative care needs, experiences and its utilisation that included some Pacific patients and families although the focus of such studies was not on Pacific patients. The limited studies on palliative care that included some Pacific participants in NZ report challenges in access and utilisation of palliative care including: lack of awareness of the role of hospice and palliative care services, language barriers and differences in cultural norms and values [15–17]. Pacific populations are also more likely to reside in socioeconomically deprived areas with worse access to a variety of health services, including palliative care [18].

At no other time are cultural identity, practices, beliefs and values more important than when someone’s health is threatened or they are approaching end of life [14]. We subscribe with the consensus from international studies that highlight inadequacies of resource and the need for culturally safe palliative care in indigenous populations [11–14] and the growing recognition within NZ of the lack of access to hospice services by Pacific and Maori patients [19]. Failing to address cultural understandings of end-of-life issues can result in care or lack of care causing unnecessary health outcomes, poor interactions, communication and lack of trust in health services. Given the above situations it is critical to explore the needs of Pacific populations in NZ requiring hospice and palliative care services.

This study’s overall and long term goal is to identify strategies to improve the provision, access and use of specialist palliative care at hospice level and to a lesser degree the generalist palliative care at the primary care level often in collaboration and through referrals from the specialist palliative care services among Pacific peoples in NZ. The specific objectives of the study towards achieving the stated long term goals were: i) to determine the utilisation by Pacific people of specialist palliative care; ii) to explore the perspectives and experiences of Pacific hospice patients and patients families, hospice care providers and relevant service providers involved in the care of Pacific populations on challenges and enablers for the use of palliative care by Pacific populations. The findings from the study will submitted to appropriate stakeholders and contribute to achieving the study’s overall long term goal.

## Methods

### Study design

This is a qualitative analysis of interview data of hospice patients and their families, hospice service providers, and other health care providers. The interviews conducted were semi-structured, face-to-face interviews, and explored the various perspectives of these stakeholders on the enablers and barriers of the uptake of hospice and palliative care services among Pacific populations in NZ. The study received ethical approval from the Health and Disability Ethics Committee of the Ministry of Health of New Zealand 16/CEN/63 (02 June, 2016). The interviews were conducted between November 2016 and January 2018.

This (qualitative) approach was chosen as it is consistent with the philosophy of palliative care: promoting the care of the whole person through a multidisciplinary approach and the contribution of qualitative research to care at end-of-life [19]. Also, it explores questions that are not easily quantifiable and culturally complex including attitudes, values and beliefs. The interview questionnaire guidelines (Interview Questionnaire Guidelines) was designed on the basis of results from international [14] and NZ studies [16, 17] and discussions with hospice care service providers, Pacific key informants and Pacific care providers.

The study was undertaken in collaboration with two hospice service providers in the Auckland region (Mercy Hospice and Totara Hospice) and one in the Wellington region (Mary Potter Hospice); both regions are where 78% of New Zealand’s Pacific people live. Pacific patients for example are the leading minority populations served by Totara Hospice.

### Participants

Eligible hospice patient participants (*n* = 7) were those: (a) whose ethnicity were registered as a Pacific islander at the above collaborating hospices; (b) were deemed physically and mentally capable of participating in an interview in a meaningful way and free from a diagnosis of dementia (based on the hospice health staff professional knowledge of individual patients). Recruitment were through the staff of the collaborating hospices. Family member participants (*n* = 15) were identified by the hospice as being the main carer of the patient at the household and having been established as the family and patient’s main contact with the hospice; the potential participants were recruited by telephone. Hospice care provider participants (*n* = 4) were identified by the collaborating hospices and recruited face-to-face by the research team. Mainstream health services providers (health providers not working as employees of hospice) (*n* = 7) and Pacific key informants (personnel identified by Pacific people as key personnel and primarily of Pacific ethnicity who are familiar through their associations and leading roles in the Pacific communities with a sense for the communities social issues, interests, activities, cultural and related socio economic perspectives.) (*n* = 11) were purposively sampled with assistance from Pacific health providers and through Pacific community networks. GPs (*n* = 3) were identified among the limited GPs in Auckland and Wellington of Pacific ethnicity or working practices with a high Pacific clientele. All participants were given a written information sheet about the study and given instructions on how to contact the research team if they required further information. All of the participants were over 18 years old and all were provided with a paid voucher of their choice for either transport or shopping in acknowledgement and appreciation of their time and effort.

### Data collection and analysis

Pacific participants were offered a face-to-face interview in a Pacific language and location of their choice. All participants elected their interviews to be conducted in English. Eight patients were recruited, one did not end up going through with the interview due to medical reasons, resulting in seven patients completing the interviews. All seven patients’ interviews were conducted at their homes. All family members interviews were also conducted at home. Key informants and health provider interviews were conducted at their place of work except of one was done at home. There were no declines from the participants who were requested for interviews. The interviews were conducted by either the principal investigator and/or a trained interviewer involving primarily open-ended questions exploring broad areas related to the patients’ and family members’ perceptions, experiences, and understanding of the goals and objectives of hospice and palliative care services. Health and hospice providers were asked about their perspectives on hospice and palliative care with a focus on what is and what is not working, and suggestions for improvement.

Informed written consent was obtained in person from participants prior to the conduct of interviews and participants understood that the interviews were audio-recorded and subsequently transcribed verbatim. The transcription was carried out by Full Stop Digital Production of Wellington, with confidentiality agreement. Data collection stopped when data saturation (when no new information is emerging from the interviews) was achieved [20]. The data was analysed using thematic analysis [21]. The following steps were taken in the analysis: i) thoroughly reading and re-reading of the transcripts and ii) noting down initial ideas independently by the principal investigator and one interviewer. All the transcripts were analysed together. The two team members then discussed and began generating initial codes across the entire data set and collating data relevant to each code and potential themes that had arisen through the coding. Transcripts were uploaded into the qualitative software NVivo 12 and each was coded according to the identified themes. The themes were then reviewed and discussed further by the team to determine the relevance of the data grouped by theme.

## Results

A total of 45 face-to-face interviews were conducted. The characteristics of the participants are described in Table 1.

**Table 1:** Characteristics of participants

Characteristics of participants	Number	Percent
**Gender**		
Female	32	71.1
Male	13	28.9
**Ethnicity**		
Pacific	42	93.3
Non-Pacific	3	6.7
**Role**		
Family member	15	33.3
Hospice patient	7	15.6
Hospice Care Provider	4	8.9
Mainstream health care provider	8	17.8
Key informant	11	24.4
**Familial member relation to hospice/palliative patient**		
Son or daughter	10	45.6
Husband	1	4.5
Wife	2	9.1
Daughter-in-law	1	4.5
Sister	1	4.5
**Mainstream health care provider characteristics**		
**Gender**		
Male	1	12.5
Female	7	87.5
**Professional Role**		
Palliative Care Specialist	1	12.5
General Practitioner	3	37.5
Nursing personnel	4	50
**Ethnicity**		
Pacific	6	75
Non-Pacific	2	25
**Diagnosis of Patients**		
Cancer	5	71.4
Motor neuron disease	2	28.6

Five main themes emerged from the data:

- Perceptions of hospice and palliative care services;
- Families’ role to look after their own and sick elderly
- Experiences with hospice and palliative services;
- Continuity of care in the community;
- Information and communication;

Key themes and related categories and quotations are outlined in Table 2.

**Table 2:** Quotations and themes

*Quotations*	*Categories*	*Themes*
*First of all … there is no description or word in our Pacific Island that … describes Palliative Care (Int 5: Hospice Provider)*	*A matter of context*	*Perceptions of hospice and palliative services*
*I heard of hospice before, I really thought it was just the shops you know, where this clothing and things (Int 4: Family Member).*	*Lack of knowledge*	
*A common myth I’ve seen in our Pacific families is that when you give them morphine (for pain) it’s euthanasia (Int 17: GP)*	*Misconception*	
*I didn’t understand about it (hospice) until my wife was sick (Int 1: Family Member)*	*Perception*	
*People close to dying they wait there (Int 27: Key Informant)*	*Limited information*	
*You shouldn’t take your parents to the hospice but look after them cause you know they’ve looked after you for all this time … don’t leave them alone … to have taking care by other people (Int 4: Family Member)*	*Family responsibility*	*Families’ role (Looking after your own)*
*The younger generation (of Tongans in NZ) …they don’t seem to want to get involved in that whole grandmother, great-grandmother care thing. They’d rather keep their distance*	*Role of family*	
*I mean hospice is also all the way in Wellington. How can you go back and forth, back and forth when you live out here? Whose got time to do that all the time and spend as much time as you can. Sometimes it’s easier for people to keep them at home and you know because we’re comfortable with that process’*	*Logistics and location that enhances families’ role*	
*“We didn’t want the staff to tell our dad he had cancer… we wanted to tell him (ourselves) when he was ready … in fact one of the friggen nutritionist came in and she was talking about it … and we hadn’t as a family … yeah it was hard for him” (Int 3: FM).*	*Role of family in decision making*	
*For me hospice has a more homely environment, you have that bond with the staff … it’s welcoming … is free … we are living in a hotel, a luxury hotel.*	*Positive shift in views with contact*	*Experiences with hospice and palliative care services*
*It’s like eating bacon and eggs every morning*	*Satisfaction*	
*It felt to me like we’ve got this equipment but we don’t really want to give it to you if you don’t need it…. I know my dad is very sick, very, very sick bar a miracle he’s going to die … it felt like you’re withholding stuff cause you think he’s going to die … So yeah that doesn’t help.*	*Concerns about suggestions of indifference*	
*I can’t talk too long …Yeah the personal care is ok, (but) I’ve got so many people that I send them away, because it doesn’t suit me and they talk and ask too many questions, and I told them that I can’t talk (due to shortness of breath), and I have listed down there the piece of paper, what they have to do, but still they ask question and I said don’t come back I’ll send for someone.*	*Sensitivity to specific disease needs*	
*She wants me to lie beside her on the same bed but they can’t provide that. So, she told the nurse ‘Oh, we’re going to go home.*	*Subtheme: privacy, intimate/emotional needs;*	*Experiences with hospice and palliative care services:*
*As soon as we sat down in his home his face just lit up, changed, he wanted to eat, he ate so much and he wanted cups of milo, he said “I’m just so happy to be in my surroundings”.*	*Subtheme: Need for variations in environment*	
*I don’t want her (hospice staff nurse) to leave. I don’t want her to go back …as she was very comforting and like family (Int 32: Patient)*	*Facilitation through caring and affection*	*Continuity of care in the community in the long term.*
*That’s the danger of us doctors and nurses and pharmacies, we think pills and medicine (Int 7: GP).*	*Polypharmacy*	
*Sometimes my mum won’t want to take it or our routine gets mixed up because we’re out and maybe she forgets to take it or she just doesn’t want to take it yeah (Int 6: FM)*	*Inappropriate and burden of multiple medications*	
*“because it takes for so long and they are paid by the head not by the time spent with the person … first, they’re slow in response, two they don’t know what they’re doing and three they forget a lot of things. … and you have to pace it. So they (GPs) try and wing them away from their practice rather than keep them” (Int 16: GP).*	*Health provider perceived client preferences*	
*as soon as they (hospice support staff) started talking to dad in Samoan, immediately there was a connection, … fantastic but there were no Pacific clinicians, … it would have been quite nice to have someone at the clinical level (Int:30 FM)*	*Appropriate format and mode of communicating*	*Information and Communication*
*“Even though, even though we talk and I say yes, bla bla bla, but maybe there’s something I’m not understood” (Int 22: Patient).*	*Ensuring adequacy and comprehensive information is delivered*	
*he/she might be working day shifts, (so) you can’t just deal with who is in the room, … know the family and look out for the person who is actually going to lead … everyone else is going to come and go but there’s going to be that one person that takes responsibility. (Int 17: GP).*	*Establishing connecting with key personnel*	
*“It’s just the caring thing … the main reason why my late wife passed away fast because she fell over there … That’s because of, I don’t know” (Int 1: FM).*	*The detailed recording and communicating of critical/and medical events*	

### Perceptions of hospice and palliative care services

It was evident from the patients/family member participants that prior to first contact with hospice care and palliative services there was a lack of familiarity with the philosophy, goals and activities of these services. According to a hospice social worker, among the major barriers to Pacific people accessing and understanding hospice and palliative care services is awareness and the need for discussions, however: “*first of all an understanding that there is no description or word in our Pacific Island that I am aware of describes Palliative Care”* (Int 5: Hospice Provider (HP)).This results in difficulties in initiating discussions when people need a number of sentences to describe palliative or hospice care services. As one family carer said: *I heard of hospice before, I really thought it was just the shops you know, where this clothing and things (*Int 4: Family Member (FM)*).* A hospice patient relates often driving past the hospice thinking: *“…it’s just for the elderly residents”* (Int 42: Patient).

In addition, the limited information available to many Pacific potential patient and family members was often inadequate and misleading with a number of Pacific participants having viewed hospices as a place associated with euthanasia: “*A common myth I’ve seen in our Pacific families is that when you give them morphine (*for pain relief*) it’s euthanasia”* (Int 17: General Practitioner (GP)). Some Pacific members were also uncertain about payment for hospice services, who is entitled to these services, whether family members can stay with patients or if the pastor can visit.

Family member participants, however, described a gradual shift in their perceptions of hospice services towards a generally more positive view, particularly after having received hospice services or through information provided by families or acquaintances that had received hospice care: *I didn’t understand about it* (hospice*) until my wife was sick* (Int 1: FM).’ The shift in perception following hearing about hospices are nevertheless limited: *“people close to dying they wait there”* (Int 27: Key Informant (KI)).

A specific question about Advance Care Planning (ACP) revealed very limited understanding of the aims and processes involved in ACP among patients and family members. One participant wondered if the ACP was just the equivalent of a will. The health providers’ views were mixed with some stating they sometimes discuss ACP with patients. However, another questioned the rationale for ACP: *I find it weird when they say to a person how do you want to die, … it feels like to me it’s taking God’s role out of it, ... it feels like they’re just wanting to put a time limit on it’* (Int 17: GP*).*

### Families’ role (looking after your own)

A dominant theme that emerged was that looking after your own elderly or family members is a fundamental family obligation, more so when they are severely ill. Hospice is also seen family members as avoiding the shared family responsibility as well as a cause for embarrassment: *“… you shouldn’t take your parents to the hospice but look after them cause you know they’ve looked after you for all this time … don’t leave them alone … to have taking care by other people”* (Int 4: FM). Putting one’s elderly and family member with severe and terminal illnesses in a hospice is viewed as: *“…you neglecting your, your responsibility:”* (Int 28: KI). On the other hand a Pacific general practitioner is of the opinion that: “*The younger generation (*of Tongans in NZ*) …they don’t seem to want to get involved in that whole grandmother, great-grandmother care thing. They’d rather keep their distance”* (Int 16: GP).

The general view among family participants and patients is that keeping and looking after one’s elderly and terminally ill family members at home keeps families closer together during challenging times, enabling extended family collective practices such as ‘sleeping over’. It also facilitates the sharing of care through teamwork and consultation processes to reach consensus and common goals including pastoral care and mental support. According to a Pacific Community Health Provider (PCHP) nurse, looking after your own at home brings services to a single location saving time and transport costs to and from palliative and health related support services, which may not be easily accessible by all family members:

*“I mean hospice is also all the way in Wellington. How can you go back and forth, back and forth when you live out here? Whose got time to do that all the time and spend as much time as you can. Sometimes it’s easier for people to keep them at home and you know because we’re comfortable with that process”* (Int 24: PCHP).

Looking after your own was also seen as ensuring a direct involvement in determining the appropriate timing of critical management processes often seen as the role of health workers; such as the appropriate timing of the announcing of a terminal outcome or diagnosis.

*“… we didn’t want the staff to tell our dad he had cancer… we wanted to tell him* (ourselves) *when he was ready … in fact one of the friggen nutritionist came in and she was talking about it … and we hadn’t as a family … yeah it was hard for him”* (Int 3: FM).

### Experiences with hospice and palliative care services

The perspectives of family members and patients (often through their families) and experiences at hospice and palliative care services were generally positive. Receiving hospice care by participants led to alleviation of certain concerns and unknowns, as well as building of trust and fostering relationship in a less ‘technical’ environment: *“…for me hospice has a more homely environment, you have that bond with the staff … it’s welcoming … is free … we are living in a hotel, a luxury hotel”* (Int 1: FM)’. Both patients and family members voice their preference for the ‘intimate’ and welcoming change from their experience with large impersonal hospitals with the quieter hospice environment spaces that is more conducive to spending time with their loved ones. Family member participants welcomed efforts by hospice staff to make families feel welcome and to encouraging them to participate in activities, morning teas and, in some instances, craft making, as well as how the hospice nurses: “*… were really kind and good and the way they handled my dad was awesome”* (Int 8: FM). An exceptionally positive experience with hospice was also conveyed by a hospice patient with terminal lung disease: *“It’s like eating bacon and eggs every morning!”* (Int 34: Patient).

However, other experiences were less positive, including suggestions of staff indifference to the patient’s comfort:

*“It felt to me like we’ve got this equipment but we don’t really want to give it to you if you don’t need it…. I know my dad is very sick, very, very sick bar a miracle he’s going to die … it felt like you’re withholding stuff cause you think he’s going to die … So yeah that doesn’t help”* (Int 3: FM).

While most aspects of personal care may be satisfactory, there are occasions where palliative care support personnel appear to ignore if not minimise the patient’s individual preferences given their condition leading to awkward situations. As one patient relates:

*“I can’t talk too long …Yeah the personal care is ok,* (but) *I've got so many people that I send them away, because it doesn't suit me and they talk and ask too many questions, and I told them that I can't talk* (due to shortness of breath), *and I have listed down there the piece of paper, what they have to do, but still they ask question and I said don't come back I'll send for someone”* (Int 2: Patient).

A sub-theme was the private and emotional needs towards the end of life that goes beyond alleviation of physical pain, as illustrated by this widower’s account of his late wife’s wish and request to simply be held close to him in her last days: “… *that’s why she wanted us to go back home from the hospice because she wants me to lie beside her on the same bed but they can’t provide that. So, she told the nurse ‘Oh, we’re going to go home’”* (Int 1: FM).

The need to reconnect with one’s home and establish a homely and family-like environment was said to be critical and contributed to positive outcomes through the psychosocial and physical interactions in patients’ experiences:

*“He thought he was going to die, he hadn’t eaten for a few days … as soon as we sat down in his home his face just lit up, changed, he wanted to eat, he ate so much and he wanted cups of milo, he said I’m just so happy to be in my surroundings”* (Int 25: HP).

On the other hand, one patient preferred the peace and quiet environment of hospice services, which can sometimes be difficult to achieve at home.

### Continuity of care in the community in the long term

Co-ordination and continuity of care in the community emerged as another major theme, with a broad range of views presented. According to a female hospice patient*:* “*I don’t want her (*hospice staff nurse*) to leave. I don’t want her to go back* …*as she was very comforting and like family”* (Int 32: Patient). Other views raised some challenges in the continuity of care at the community. Challenging issues discussed included: difficulty in navigating the myriad of professional, administrative and support personnel; and processes involved the pathway from hospice to primary level care level. Participants felt that adequate training of family member carers was vital to the continuity of an acceptable standard of care at home. Adequate training for family carers also gives them the confidence they are not doing harm while administering care or medication. Some patients who have been advised of the need for continuing palliative services through a hospice service environment are said to have been reluctant to accept such advice for ‘fear’ of hospices: *“… we are happy to see you but not hospice”* (Int 44: Palliative Care Specialist). A lack of coordination and communication had been raised regarding different specialists prescribing medications to patients without collaborating with other specialists looking after the same patient. This had been suggested to contribute to polypharmacy and potentially inappropriate medications (PIM) at the primary care. According to one GP, she often has to contact specialist to discuss what medications she can get rid of: “*that’s the danger of us doctors and nurses and pharmacies, we think pills and medicine”* (Int 7: GP). The amount of medications was said to also contribute to confusion at the individual patient level: *“sometimes my mum won’t want to take it or our routine gets mixed up because we’re out and maybe she forgets to take it or she just doesn’t want to take it yeah*” (Int 6: FM).

One health provider suggested that a lot of GPs are not keen on taking elderly Pacific clients, some of whom are palliative cases, for various reasons including financial and language barriers, and they try and encourage them away from their practice:

*“… because it takes for so long and they are paid by the head not by the time spent with the person…. he walks through the door for about 15 minutes …for old people, you’re looking at half an hour …. first, they’re slow in response, two they don’t know what they’re doing and three they forget a lot of things. … and you have to pace it, so they are not the clients that the GP’s look for to have in their practice. So they try and wing them away from their practice rather than keep them”* (Int 16: GP).

Being a palliative care patient, regardless of the duration and whether the care is carried out either at home or in hospice, may not be the full experience for the patient or the family; associated social, emotional and physical issues may be important as well, but not adequately addressed. In addition, loneliness, and the realisation of dying, can be taxing mentally in the hospice patients’ journey: “*and the mental issues not addressed adequately …That’s where I think euthanasia, some people just go … just put me to sleep… I don’t know if anyone to be honest ever asked that question, how are you feeling with all of this?”* (Int 17: GP). Participants further raised the need for clear and appropriate channels for families to engage effectively with various health support services to avoid confusion and unnecessary stress at critical times, and after hours.

### Information and communication

A general consensus among participants is the need for accessible information that is adequate, consistent and accurate. According to one hospice patient, prior admission as a hospice patient, she: *“never heard about the company … no idea”* (Int 20: Patient). In turn, families and patients who had been recipients of hospice services played a critical role in changing attitudes towards a more positive impression of hospice and associated palliative services: “*it took me two years to finally agree to go to hospice ... and I tell you, once I try I never stop going there*” (Int: 39 Patient). Pacific community health providers stressed the need for education on hospice services targeting Pacific communities. The importance of such education to be conducted in a culturally appropriate approach and format preferably by Pacific staff was emphasised by a hospice cancer patient’s son: “… *as soon as they* (hospice support staff) *started talking to dad in Samoan, immediately there was a connection, … fantastic but there were no Pacific clinicians, … it would have been quite nice to have someone at the clinical level*” (Int:30 (FM). Another hospice patient stressed the need for information and communication in a language that is best understood by patients and family members: *“Even though, even though we talk and I say yes, blab bla bla, but maybe there’s something I’m not understood”* (Int 22: Patient).

The uncertainty around entitlements for hospice services, often as it relates to one’s residency or citizenship status contributes to Pacific people ‘standing back’ resulting in a passive approach to seeking hospice and related health services. Likewise, entitlements for families to visit loved ones needs to be better communicated if not managed. A family being restricted to visit a dying son through instructions by the son’s partner can be stressful: *“this is insensitive we’re his family, we’re not going to have time limits, and so the* (hospice) *nurse said well I’m sorry his partner has decided that”* (Int 36: FM).

Participants emphasised the importance of health providers taking time to explore and understand culturally appropriate modes and family channels of communication, and the appropriate times to raise and discuss certain topics such as those of a sexual nature or bodily functions that may be culturally inappropriate to discuss with both male and female relatives present. Participants further noted that many Pacific families work multiple jobs and that, as a consequence, the person that is the ultimate decision maker, and critical to communicate with, hardly meets the hospice staff during ward rounds:

*“…he/she might be working day shifts, (so) you can’t just deal with who is in the room, … know the family and look out for the person who is actually going to lead … everyone else is going to come and go but there’s going to be that one person that takes responsibility”* (Int 17: GP).

The individual(s) making major decisions may be an extended ‘elder-wiser’ family who may not necessarily be residing in the household but could be a relative outside NZ in a distant Pacific island.

A lack of clarity of links between care, health events including serious medical outcomes or death can lead to questions of trust. This may arise due to family members’ interpretation of causes of events and whether treatments were suitable at the time. This is especially the case if family members were not present when a serious event occurs: *“It’s just the caring thing … the main reason why my late wife passed away fast because she fell over there … That’s because of, I don’t know”* (Int 1: FM).

## Discussion

Five themes were identified from the data: perceptions of hospice and palliative care services; families’ role is looking after their own sick and elderly; experiences with hospice and palliative services; continuity of care in the community in the long term; and information and communication. These five themes are interrelated and can be supported by the existence of specialist palliative care services at hospices, generalist palliative care at the primary health care level to compliment Pacific collectivism and family approach to health decisions. The health system and hierarchy of health professionals need to acknowledge and appreciate the role of the family in caring for the elderly and those at the end-of-life and appreciate the need for communication in a culturally appropriate context. This will also reinforce the reality that palliative care is an important component of comprehensive health care more generally.

There is limited literature on Pacific hospice patients’ and carers’ perceptions on hospice and palliative care services in NZ. This study showed there are notable gaps in information and knowledge on hospice and palliative services’ goals and core activities. This is consistent with previous work by Frey and colleagues, who also reported a general lack of awareness among Pacific and Māori [16, 22] participants on the range of easily accessible services associated with hospice care, as well as misconceptions about eligibility to hospice services [17]. The uncertainty around eligibility as related by participants contributes to ‘standing back’ resulting in a passive approach to seeking hospice and related health services.

Pacific traditional and cultural family practices could be better integrated into information about hospice and palliative care to enable outcomes that are more effective for Pacific families [14]. For example, “looking after your own” elderly and critically ill family members as a paramount family obligation was a prominent theme. Furtherance and empowerment of families and family carers’ confidence to actively participate in the appropriate care of their own through better information, community education and resourcing is likely to improve engagement with service providers and give better satisfaction with the use of services and possibly better outcomes for patients [15, 16]. A vital issue is continuity of care from specialist palliative care to general palliative care services and through to primary care level. This needs accounting for Pacific views on health and terminal and supportive health care, thus aligning with key principles of palliative care including cultural considerations, choice of site of care, and communication [23].

Key players in imparting information and contributing to a shift in Pacific populations perceptions of hospice care are Pacific hospice patients and their families who are often willing (if unknowingly) ‘community educators’ that would be valuable partners to address the community gap in culturally appropriate education on hospice services [16]. Such community education has the potential to alleviate some misconceptions identified by participants. A particular example is administration of pain relief, such as morphine, as tantamount to euthanasia. In Pacific populations collectivism is a basic attribute, families are central, and personal relationships are highly respected. Equally important during planning for community consultations/education and implementation is to acknowledge that within the collective there are one or more individuals who are ‘in charge’ and make final decisions in critical situations such as end of life, preparing for the dying, ACP decisions, the dying process and bereavement; all on behalf of the immediate family and the collective [14]. The interview guidelines included a specific question on the participants’ understanding and utilisation of ACP. The study found that apart from health providers, most of the other participants showed hardly any meaningful awareness of ACP. Our findings are in line with an evaluation of the Advanced Care Planning Programme [10] which found that the wide range of resources for training and information on ACPO are in English and the need for resources in Pacific languages; district health boards undertaking community programmes but engaged less with Pacific communities. In instances where ACP Coordinators followed up patients to review ACPs, some Pacific consumers could not recall having conversations around end of life planning or developing plans, further suggesting that conversations and resources were not culturally appropriate for consumers [10]. The health providers on the other hand indicated either a lack of active promotion of ACP due to busy schedules with one questioning the need for ACP based on moral and individual beliefs. The above evaluation also found although primary care services sits across the ACP pathway, competing demands and the time it takes to develop a plan especially in a challenge in any general practice setting. At the same time the role of the community is vital and untapped in promoting ACP through building of relationships over time in a culturally appropriate context [10], with consumers actively involved in writing their own plans at homes; with someone they had a relationship with and with family involved.

In a managed care climate, where palliative care patients contact is often with multiple health and medical specialties, patients will increasingly see health care providers from cultural backgrounds other than their own. The differences in beliefs, values and traditional health practices are of particular relevance at the end of life and may compromise continuity of care [24]. Specialists not ‘talking to each other’ on medication management of individual patients is a barrier to the efficient implementation of palliative care, as well as not helping with the patient’s health literacy. The requirement for proper discussion on limits of what treatment can achieve, and the specialist informing the GP adequately was identified as potentially leading to PIM. These findings are in line with those from previous research which emphasised the need for improved inter-professional communication, to address PIM, especially in palliative care [25–30].

A robust and systematic process to elicit, record, and integrate the patients’ expertise/experience, preferences and priorities about medications creates an ongoing space for patients or caregivers to participate in shared decision-making. The critical role of primary care providers and their input at the primary care level and long term is critical and needs recognition from a policy level perspective and reinforce palliative care as an important component of comprehensive health care [19]. Families agreed that primary care should assist with encouraging Pacific people to use hospice and palliative care services, and hospice workers cited that a more sustainable increase in understanding of hospice and palliative care services can only take place through a strategic regional, rather than individual level.

Death and terminal illness are understood and experienced within a complex web of cultural contexts [31] and the care for the terminally ill that lacks cultural sensitivity may result in unwanted outcomes, poor interactions and loss of trust in health care services in general and health professionals [32]. Hence, the need for robust and systematic open discussion and education of patients/families on medication use, potential side effects, treatment priorities and the detailed documentation of medical and physical events. Addressing the relative lack of Pacific people in the palliative care and hospice workforce and those who speak Pacific languages has been shown to help patients and their family feel safe and comfortable [5], as well as enriching any cultural contexts. A sub-theme identified is a perceived lack in addressing the mental and emotional needs including loneliness and social isolation among patients. These resonate with other studies involving Pacific populations in NZ [33] which described experience of social isolation and loneliness and the need for more culturally appropriate services, greater mental-health support and more service provision on weekends and evenings. Another sub-theme identified is the need for a change of environment such as a ‘break’ from hospice environment preferably to re-visit one’s home or familiar environment.

The notion of ‘home’ is well known in everyday life and its positive connotation in relation to palliative care and issues related to end-of life and dying [34, 35]. These include the ethnic and spiritual need to ‘come home’ by returning to one’s Pacific island of birth once curative treatment is ceased and mortality accepted. In that while dying means leaving life or hospice it is going on a journey and ‘coming home’ [34]. The literature talks about the elderly being reluctant to move to new environments although often forced to do so either for health care or migration. For them, a home plays a critical role in maintaining a sense of personal identity (in this case one’s place of birth in the Pacific for example) and independence, sustaining a meaningful existence, and resisting institutionalization [36]. However, the physical and emotional transformation of a home visit, or the decision to go home as the last alternative to achieve a dying wish, such as lying next to a loved one in the same bed, is often not possible at a hospice. Participants acknowledge that going back to one’s ethnic ‘home’, has significant social and financial implications; in particular, families may be separated, as well as the associated practical and cost barriers to international travel for sick individuals, with limited availability of hospice or palliative services in the preferred Pacific islands destination. Also, leaving NZ to reside in another Pacific island country means certain disability allowances being discontinued as well as one’s NZ Superannuation being reviewed for continuing full payment or payment for only part of what one is entitled to. Among the few exceptions to this rule is Niue which has an ‘Aged Ward’ in its hospital that provides some palliative services as well as patients continuing to receive any NZ pensions and entitlement allowances while residing outside NZ. The participants suggested that a win-win situation from a socio-economic perspective for both the NZ services and involved families is feasible if similar ‘portable’ pensions be applicable to other Pacific islanders returning ‘home’. These warrants exploring at the policy level, given it has implications for the promotion and establishment of much needed quality hospice and palliative care in the wider Pacific island region.

The vision of The New Zealand Palliative Care Strategy [37] is for “All people who are dying and their family/whānau who could benefit from palliative care services have timely access to quality palliative care”. Specific populations in New Zealand are recognised as facing inequality in accessing and utilising hospice and palliative care including Maori and Pacific peoples [10]. Pacific people generally have a wide range of health beliefs and in particular towards end of life care. Palliative care through hospices or primary care level therefore provides an opportunity for a “Pacific meets West” platform of medical care as Pacific people have little option but to interact with so called Western health practices. Promoting and seeking Pacific population’s views on palliative care is an opportunity for further communication, education and understanding to address the lack of awareness and certain misconceptions about hospice and palliative care goals and objectives. The findings highlight the need for incorporating a whole of health strategy through the evidence based knowledge within palliative care and the cultural lens of Pacific such as collectivism and the role of the family in care giving. More importantly, hospice and palliative care complements therapies that aim to cure or control disease processes throughout an illness and not just at the end of life.

### Limitations

Patients’ and families were already engaged with the hospice and may be less likely to have experienced barriers to accessing this service and is a potential study limitation. Nonetheless, the participants also discussed their experiences prior engaging with the hospice and palliative care services as well as discussing their experiences with the services they are receiving and suggestions for improvements. The study was carried out in only three hospices and while the authors cannot generalise this study finding to other Pacific populations it is also important to note that over 78% of Pacific people in NZ live in the two regions where the study was conducted (Auckland and Wellington). The interview schedule specifically asked for the participants’ understanding of hospice, its goals, objectives and services. The participants were also asked about their understanding of palliative care and in terms of palliative care provided by the hospice but not palliative care as a management approach.

## Conclusion

Pacific people do not optimally benefit from hospice and palliative care services as currently available. In part this is due to a lack of awareness of the philosophy and practices of hospice and palliative care services, and partly due to current health services that are culturally orientated to autonomous and informed individuals.

There is thus a clear need for more educational programmes for Pacific populations on the philosophy, goals and access to such services as well as the need for the hospice to ‘come home’ either in the household or a Pacific cultural framework and context, incorporating the themes identified from this study.

## Supplementary information

**Additional file 1.** Interview Questionnaire Guidelines.

## Data Availability

The datasets used and/or analysed during the current study available from the corresponding author on reasonable request.

## Abbreviations

ACP: Advance care planning
CRD: Chronic respiratory diseases
CVD: Cardiovascular diseases
DHB: District Health Board
FM: Family member
GP: General practitioner
HP: Hospice provider
KI: Key informant
NZ: Aotearoa/New Zealand
PCHP: Pacific community health provider
PIM: Potentially inappropriate medications
