# Supplementary material for: Pacific meets west in addressing palliative care for Pacific populations in Aotearoa/New Zealand: a qualitative study
Source: BMC Palliat Care. 2020 Jul 8;19:100. doi: 10.1186/s12904-020-00604-2 (PMC7346658; doi:10.1186/s12904-020-00604-2)
Supplement: Supplementary file 1 — Additional file 1. Interview Questionnaire Guidelines. [file 12904_2020_604_MOESM1_ESM.docx]

**Pacific meets West in Advancing Palliative Care for Pacific populations**

**Hospice Care Provider Interview Guideline**

1. Can we start by you telling me/us about you…and about your Organisation and what is your role with the hospice?

2. How are people referred to your hospice

3. Can you identify known strategies that have been recently used to improve hospice care access and utilization for Pacific people, both in your organization and more broadly?

*Probes: examples, any changes and how was it changed?*

4. If there were strategies as above then, what are challenges/ or things that are/were helpful to implement the above strategies (used in your setting to improve palliative care in the hospice and outside for Pacific people?

*Probes: elaborate, influences daily work, influencing factors*

5. So, what are you going/would you like to do next in your service?

*Probes: why this strategy, consequences, other strategies that may be needed*

6. What other supports is being provided to help with the patients?

*Probes: communication, trust, long term relationship with primary health*

*providers, family, spiritual needs, traditional practices, workforce, hospice relationship with other organizations*

8. Have you discussed Advanced Care Planning with some of the patients?

*Probes: If no why, understood? If not understood, give one line definition. If yes how do families/patients respond*?

9 We have discussed Pacific people and Hospice and Palliative Care, are there areas that you think that could be improved overall that affects not only Pacific but the general population as a whole?

10. Is there anything else you would like to say and discuss which we have not asked you about?

Thank you for taking part in this study. We are grateful for your time and sharing your experiences so that we can find out more about hospice care experiences for Pacific people.

………………………………………………………………….

**Mainstream Health Care Provider Interview Guideline**

1. Can we start by you telling me/us about you, your organisation, services provided and your role in the organization (clinic/hospital/health provider etc)?

2. Do you have direct/indirect collaborating with hospice services? Palliative care in general?

*Probe: who do you mostly see from the hospice, doctor/nurse/social worker, others? How often do you see them if so, how often and is this enough?*

**3.** What kinds of things does your organization do to help whanau/families in hospice care?

**4.** What do your clients think about hospice services/palliative care?

*Probe: Palliative care as part of collaboration with hospice services, palliative care in general*

5. Do you think the hospice and your organization’s communication are adequate and appropriate?

*Probe: why? Elaborate*

6. If your hospice clients or palliative need patients need help after hours (after 5pm and before 8am) do they have knowledge of who to call?

7. Have you talked to your patients/potential or palliative care patients about Advanced Care Planning?

*Probe: If no and what is it –ONE LINE DEFINITION, if yes, what did they say, how did they feel about advanced care planning?*

8. Are there any areas in hospice care or palliative care services where you think things could be better for your patients or overall?

9. What would your clients and their family like to happen in their palliative care’?

*Probe: communication, family, spiritual needs, respite. Medication, traditional practices*

10. Is there anything else that you think we should discuss which I have not asked about?

Thank you for taking part in this study. We are grateful for your time and sharing your experiences so that we can find out more about hospice care experiences for Pacific people.

……………………………………………………

**Hospice Patient/Family Member/Carer Interview Guideline**

1. I hope you don’t mind by us starting with you telling me/us about your understanding of the goals, objectives of hospice services and the palliative care services they provide? ***Probes****: Who first told you about hospice care? How were you introduced to hospice? (support for referral process, meeting with hospice nurse/doctor/social worker for the first time); views and understanding of philosophy of hospice prior engaging with hospice and after?*
2. Can you tell me how long have you been looked after by hospice? What did you know about the work that they do prior engaging with hospice services? ***Probes****: specialist/generalist palliative care, looking after families, shared care, respite care, care at home, inpatient unit)*
3. Who do you mostly have contact with from the hospice? (hospice nurse, social worker, doctor) How often do you see them? Do you feel that is enough? Are you happy with the way that they look after you? ***Probes:*** *why, why not, cultural safety, communication?*
4. Do you have a regular GP or primary health care provider? Do they (doctor, district nurse, community worker) keep in regular contact with you? (In what ways?)
5. Do you know who is in charge of your care? Do you think the different people from hospice/your GP communicate with each other about your care? (In what ways?)
6. Are there any differences in the way you are cared for now compared to before hospice? ***Probes****: communication, family, spiritual needs, respite, clinical/medication, traditional practices*
7. Are there things that you think could be better for Pacific people’s hospice/palliative care? ***Probes****: communication, family, spiritual needs, respite, clinical/medication, traditional practices*
8. Who provides support for your carer/family members? How is this provided? ***Probes****: church, community, hospice social worker/counsellor, hospice services such as art therapy classes?*
9. What is after hours care like for you now? (do you have a number to call, what would happen if you call the number –phone support or a visit) How does this compare with after-hours care before hospice?
10. Is there anything else you would like to say about your care? Anything that you think could be done better? ***Probes****: communication, involvement of family, physical care, religious and traditional practices, food, people who provide the care*

***………………………………………………………..***

**Key Informant Interview Guideline**

1. Can we talk a little about you? (ethnicity, interests,)
2. Currently working? (If so, current occupation, services they provide?)
3. Do you or (if employed) your organization or organization such as a community or voluntary/church etc are actively involved in engaging with or is helping families in hospice care?
4. Would you have some idea/thoughts on some of the reasons for Pacific people either utilising or not willing to utilise hospice services if they are free or economically affordable? ***Probes****: What would be some of the ways of addressing the problems (if any) you refer to?* ***OR*** *What would be some of the ways of complimenting and sustaining/ further promoting and increasing the use of hospice services among current users and their whanau?*
5. Who are other personnel or organisations that you think I should talk to who would have in-depth knowledge or views on the access and utilisation of hospice services by Pacific?
6. Have you heard of or know something about or called Advance Care Planning?

Prompts: If no and what is it – Give a one-line definition

1. Is there anything else that you think we should discuss which I have not asked about?

Thank you for taking part in this study. We are grateful for your time and sharing your experiences so that we can find out more about hospice care experiences for Pacific people.

**………………………………………………**
